# “I have got diabetes!” – interviews of patients newly diagnosed with type 2 diabetes

**DOI:** 10.1186/s12902-019-0380-5

**Published:** 2019-05-24

**Authors:** M. Pikkemaat, K. Bengtsson Boström, E. L. Strandberg

**Affiliations:** 1 Husensjö Health Care Center, Skaragatan 102, S-25363 Helsingborg, Sweden; 2 Center for Primary Health Care Research, Department of Clinical Sciences, Lund University, Malmö, Sweden; 3 R&D Center Skaraborg Primary Care, Skövde, Sweden; 4 Department of Public Health and Community Medicine, Primary Health Care, the Sahlgrenska Academy, University of Gothenburg, Gothenburg, Sweden; 5 Department of Clinical Sciences Malmö, Family Medicine, Lund University, Malmö, Sweden

**Keywords:** Type 2 diabetes, Newly diagnosed, Interview, Primary health care, Complications, Qualitative content analysis

## Abstract

**Background:**

To be diagnosed with type 2 diabetes is a challenge for every patient. There are previous studies on patients’ experience in general but not addressing the increased cardiovascular risk and multifactorial treatment. The aim of this study was to explore the thoughts, experiences and reactions of newly diagnosed patients with diabetes to this diagnosis and to the risk of developing complications.

**Methods:**

Ten adults (7 men/3 women, aged 50–79) diagnosed with type 2 diabetes within the last 12 months were interviewed at a primary health care center in Sweden. An interview guide was used in the semi-structured interviews that were transcribed verbatim. The analysis was qualitative and inspired by systematic text condensation (Malterud). The text was read several times and meaning units were identified. Related meaning units were sorted into codes and related codes into categories during several meetings between the authors. Finally, the categories were merged and formed themes.

**Results:**

We defined three main themes: Reaction to diagnosis, Life changes and Concerns about the future. Most patients reacted to the diagnosis without intensive feelings. Lifestyle changes were mainly accepted but hard to achieve. The patients’ major concerns for the future were the consequences for daily life (being able to drive and read) and concerns for relatives rather than anxieties regarding medical issues such as laboratory tests. There were considerable differences in how much patients wanted to know about their future risks.

**Conclusions:**

The results of this study might help to focus doctor-patient communication on issues highlighted by the patients and on the importance of individualizing information and recommendations for each patient.

**Electronic supplementary material:**

The online version of this article (10.1186/s12902-019-0380-5) contains supplementary material, which is available to authorized users.

## Background

The prevalence of type 2 diabetes has increased in the last few decades [1], while the average age at diagnosis has decreased [2]. Diabetes confers an elevated risk of cardiovascular complications or premature death compared to the background population [3]. To lower the risk of complications it is important to lower the glucose level but also to treat risk factors such as hypertension, hyperlipidemia and obesity [4]. Therefore, starting at the time of diagnosis the patient is prescribed several drugs over a short time. Necessary changes of lifestyle are also challenging and can radically change the patient’s way of life [5, 6].

In Sweden, patients with type 2 diabetes mellitus are usually taken care of at the primary healthcare centers (PHCCs) [7]. The PHCCs are responsible for a certain number of listed patients. Both General Practitioners (GPs), mostly specialists in family medicine, and diabetes specialist nurses meet the diabetes patients. The care is based on regional guidelines based on the Swedish national guidelines [8]. In Sweden, a patient with diabetes with no complications or changes of medication, visits the practice twice a year for check-ups, which means meeting the GP once a year and meeting the nurse once a year on a routine basis. Additional visits take place only when needed, for example if complications arise or after a change of medication. Prescriptions are often renewed with four iterations to cover 1 year and can be renewed by e-mail without physical meetings with the GP. Other professionals available to the patients at the PHCCs may be chiropodists, social workers, physiotherapists, psychologists and/or dieticians. Only in case of complications which cannot be managed at the PHCC is the patient referred to other specialists [7]. The Swedish health care system is publicly financed. Coverage is universal and automatic. Private health insurance, in the form of supplementary coverage, accounts for less than 1% of expenditures.

The health care staff and specifically the physician are obliged to inform the patient about the importance of pharmacological treatment and the benefits of the lifestyle changes to reach the different goals of treatment.

Most patients understand the benefit of normalizing blood glucose levels. Discussions about blood pressure and treatment with lipid lowering treatment blood lipids can be more challenging. The target levels for blood pressure prevailing for patients with diabetes at the time of the study were set in accord with the European guidelines: less than 140/85, somewhat lower than for patients without diabetes patients [9]. Lipid target levels for low-density lipoprotein (LDL) cholesterol were less than 2.5 mmol/L for patients without additional complications or risk factors, and 1.8 mmol/L for patients with additional complications, which is lower than for patients without diabetes [8]. Consequently levels that were seen as normal before diagnosis become elevated or pathological and requiring drug treatment.

Patients may have very different views about the value of knowing about the risk of complications. The patient’s view of a diagnosis of diabetes has been studied before. Reactions can vary from individual to individual, from shock to acceptance or no worry at all. Some patients even deny or repudiate the diagnosis, while others fear the complications [10, 11].

Regarding the risk of complications there is a great deal of previous research as well as ongoing research to refine the prediction of risk and to detect high-risk patients at an early stage, including the use of biomarkers [12–15]. But it is easy to forget what risk assessment means to the individual patient, and this field has to our knowledge not been previously studied in patients with newly diagnosed diabetes, providing a gap for new research to fill.

Qualitative research including research interviews are valuable complements to quantitative research, helping us to understand individuals and to focus on their thoughts and experiences [16, 17]. Qualitative studies addressing the experiences of patients with diabetes mainly focus on prevalent complications [18, 19].

There are previous studies on patients’ experience in general but not concerning the increased cardiovascular risk and the multifactorial treatment, especially in newly diagnosed patients with type 2 diabetes. The aim of our study was to explore the thoughts and experiences of these patients.

## Methods

### Design

We chose a qualitative design and individual interviews for this project. Inspired by Kvale [17], an interview guide for semi-structured interviews was developed (see Additional file 1) by the first author, MP, who is a GP and PhD, assisted by the co-authors, KBB, GP with long clinical experience of diabetes care, and ELS, a behavioral scientist with solid experience in qualitative methods, both as a researcher and as a tutor. The guide contained open questions to stimulate the interviewees’ own story and when needed follow-up questions. The main areas were the interviewees’ experiences and thoughts about their diagnosis, the information given by the health care system, risks and complications of the disease, drug treatment and lifestyle changes.

### Participants

We interviewed adults first diagnosed as having type 2 diabetes mellitus within the last 12 months. They were patients at a PHCC in southern Sweden where the first author, MP, works as a GP. It is medium-sized, having about 9000 listed patients, of all ages and of both Swedish and foreign background. The PHCC is staffed with specialized registered nurses taking care of patients with diabetes. A list of all patients from the PHCC first diagnosed as having diabetes mellitus within the last 12 months was created with help from the patients’ charts. The author’s own patients were excluded from the study. Inclusion criteria apart from a diagnosis of type 2 diabetes in the last 12 months were: ability to participate in the interview without help, i.e. understanding and speaking Swedish, and having no cognitive impairment making the interview difficult to perform. There were no exclusion criteria apart from not meeting the inclusion criteria. The patients on the list were contacted either by the nurses or by the author, consecutively until no further patients were needed. Interested persons received both oral and written information from the nurses or from MP, and after giving written consent a date was set for the interview.

### Interviews

We planned for 10–12 interviews, based on previous studies showing this number to be adequate to achieve saturation, i.e. identifying all the main variations [20]. MP, the GP, performed all the interviews at the PHCC. The interviewees were offered a more neutral place, but the PHCC was found most convenient for them.

The interview started with general information. The GP performing the interviews presented herself and the aim of the project once more. Information about the participants’ anonymity and their right to stop the interview was repeated. Data about the participants’ age, gender, place of birth and date of diabetes diagnosis was noted.

In the following part the semi-structured interview guide was used as a support with follow-up questions when needed. The interviewees were encouraged to talk freely about their own reflections. This part of the interview was started with an open question about the participants’ thoughts or feelings about having been diagnosed as having diabetes. The participants had the possibility to interpret the question in their own way, giving them space and time to answer freely and even to change topic. Specific questions were mainly used by the GP when the participant fell silent for some time. The interviewer had the three following general topics in her mind: information provided about the disease or treatment, complications of the disease and changes in life after the diagnosis. Those topics were not asked about in chronological or structured form, but possible probing questions were asked when and if the GP felt them meaningful and needed. Such questions could be “Do you see yourself as involved in the treatment process?”, “What do you think about eventual diabetes complications?” or “Do you have any thoughts about changes in your life since your diagnosis of diabetes?” At the end of the interview the participants were asked if there was anything more they would like to add or to conclude and if they had any questions themselves, before the GP thanked them for their participation.

The interviews were recorded digitally and transcribed verbatim by a research assistant experienced in writing interview texts. The texts were then compared to the recordings by MP.

### Analysis

The method for the analysis was qualitative and inspired by systematic text condensation in four steps according to Malterud [21]. First the text of all the interviews completed was read through several times by the author and the co-authors to get an overview of the data and a general impression of the whole, with an open mind and without theoretical background knowledge or expectations. Reading for the first time was done without making any notes; the second or third time the authors started to summarize their impressions and some preliminary themes emerged, often spontaneous associations arising, similar to preliminary topics. Meeting in person, the authors discussed those and noted initially 14 preliminary themes, already having in mind that some of them might be merged later on.

In the second step “meaning units” in the text were identified by the author and the co-authors, first on their own and then in discussion when meeting. A “meaning unit” was defined as a text fragment containing information in relation to the research question. We started to classify and sort the meaning units we had detected. We marked the meaning units with a code, meaning a label that connected related meaning units. These related meaning units with the same labels formed code groups. At the same time we continued working on the preliminary themes, especially merging some of the preliminary themes. We were flexible in the coding procedure and changed both codes and classifications several times during the procedure while discussing in the group.

In the third step, also called condensation, we only used the text of the meaning units as a decontextualized selection. We worked with one code group after the other. The codes led to categories, and when necessary the categories were divided once more into subcategories.

We worked both on our own and together, discussing the importance of the different points of view, taking advantage of the different working backgrounds of the authors. The codes and categories were discussed several times. As a next step the categories were sorted and classified. Finally the categories were merged to a group of categories belonging together, i.e. describing similar information and forming a theme, which ultimately led to three definitive themes.

In the fourth step, the reconceptualization, we analyzed the content of the different categories one more time, meaning that we put the pieces together again and developed a story with the different meaning units as a base. We wrote a narrative text with our own words using particular examples from the text to illustrate the results. This was repeated for every category.

### Ethical considerations

The study was approved by the Regional Ethics Review Board at Lund University.

## Results

Interim readings of text found that saturation was reached after 10 interviews; no further interviews were performed. The interviewees were 3 women and 7 men, Table 1.

**Table 1:** Characteristics of the interviews

No.	Age (years)	Gender	Country of birth	Length of interview (min)
1	69	man	Sweden	11
2	79	man	Sweden	21
3	74	man	Eastern Europe	32
4	49	man	Southeastern Europe	17
5	50	woman	Sweden	16
6	79	man	Southeastern Europe	29
7	60	man	Sweden	45
8	71	man	Sweden	16
9	57	woman	Sweden	30
10	60	woman	Sweden	15

During the coding process ten categories emerged, when needed supplemented with subcategories. Examples of text condensation into meaning units, codes and categories are shown in Table 2.

**Table 2:** Examples of coding and categorizing, theme “Concerns about the future”

Meaning units	Code	Category
It would be unfortunate if my sight was affected because I can only see in one eye as it is today […] if that gets worse I’ll be blind in practice. I read quite a lot, so if my sight deteriorates even more it would mean a much much worse life […] I have devoted much of my activity to reading, watching television, keeping informed in general […] So if the sharpness of vision got lost I would be isolated and it would be very serious if that happened (2, 3, 14)	Fear of visual impact	Functional disabilities
What I worry about, I suppose […] my heart, I that think it has had to work rather hard and maybe it will give up some day […] if it’s damaged it’s damaged […] then I can’t influence it so much (9, 9, 5)	Fear of cardiovascular complications	
Foot ulcers are troublesome […] so I wouldn’t want that, and I don’t want to go blind either […] but foot ulcers are probably what I’m most afraid of, well, not afraid, but I don’t want them (10, 5, 20)	Fear of foot complications	
The only thing that worries me was that I would have to stop flying (1, 3, 26)	Fear of not being able to perform leisure activity	

After further discussions the categories were finally grouped into three main themes comprising 3, 4 and 3 categories: **Reaction to diagnosis, Life changes** and **Concerns about the future**, Table 3.

**Table 3:** Themes, Categories and Subcategories

Theme	Category	Subcategory
1. Reaction to diagnosis	Denial	Skepticism
		Unexpected diagnosis
	Guilt	Shame
		Disappointment
	Acceptance	Neutral attitude
		Logical consequence
2. Life changes	Being diagnosed with diabetes	Comparison with other people with diabetes
		Relation to surrounding persons
	Therapeutic treatment	Non-pharmacological treatment (dietary changes and physical activity)
		Pharmacological treatment (oral medication, insulin)
	Relationship to health care	Expectations
		Trust
	The importance of knowledge	Obtaining supplementary information about diabetes
		Relating individually to the information
3. Concerns about the future	Family	Heredity; taking care of their family
	Functional disabilities	Physical complications and their consequences
	Attitudes towards control and risk	Need for control
		Wanting to know about risks of future complications

### Reaction to diagnosis (Table 4)

Several people reacted with **denial** as they were diagnosed at an annual checkup and were not prepared, it was an *unexpected diagnosis*. Almost all individuals had no symptoms, which led to *skepticism*, and it took some time to accept the diagnosis. Some participants associated the diagnosis with **guilt**; a female interviewee talked about a huge amount of *shame* which led her to keep the diagnosis secret. Some individuals reacted with *disappointment* and grief.

**Table 4:** Categories and examples of meaning units for theme 1: Reaction to diagnosis

Category (Subcategory)	Meaning units
Denial (Skepticism, unexpected diagnosis)	Well, first and foremost there’s complete denial [on being diagnosed] because I haven’t noticed any symptoms (Interviewperson (IP) 7)
	so I’m still a bit skeptical about the diagnosis … wonder if it’s confirmed (IP7)
	I haven’t noticed anything, but because I fly I have to go to the doctor once a year and so he discovered it (IP1)
Guilt (Shame, disappointment)	[that you yourself are partly to blame] I think about these lifestyle diseases, they hit you because you have a lifestyle that’s not really okay, and then that maybe we have a society that enables the lifestyle, that’s another matter, but there’s nothing really to say that you have to adopt it (IP9)
	so this was quite a shock in a way, although in a way it wasn’t, but unfortunate … I didn’t want this (IP9)
	it’s not much fun talking about it, I hope I can stop […] it’s the disappointment about ending up in this situation (IP9)
Acceptance (Neutral attitude, logical consequence)	[having diabetes] doesn’t mean much [to me] … I’ve been through so much shit all my life, I don’t react, I live as I live (IP6)
	it’s a common process at my age that you get it [diabetes] (IP2)
	I was so prepared for [the diagnosis] and I had felt it in my body and I knew I was overweight … I knew that we have had type 2 diabetes in the family […] I knew what to recognize (IP10)

The majority, however, reacted with **acceptance**. The information about the diabetes diagnosis was met with a *neutral attitude* and the interviewees did not think a lot about it.

*“I take one day at a time … or one week at a time […] I don’t go around thinking about it … it’s just the way it is and it is going to be like this.” (Participant (P) 8).*

For some it was a *logical consequence* of their previous living habits, while others explained the diagnosis as the normal process of aging or heredity.

*“The whole body […] gets worn out like an old car […] it’s not possible to keep going forever.” (P2)*

### Life changes (Table 5)

**Being diagnosed with diabetes** changed the lives of the participants. *Comparison with other people with diabetes* was important, especially with those who had suffered from diabetes longer and needed treatment with insulin. It was important for several interviewees to dissociate from those people because they did not feel like them, nor did they want to become like them. They talked spontaneously about problems and complications other people with diabetes suffered from, such as fainting, becoming blind or dying early. Their lives were sad and complicated, for example, when traveling. In contrast, some interviewees talked about other persons who lived a good life and could take advantage of the diabetes diagnosis to receive free pedicure.

**Table 5:** Categories and examples of meaning units for theme 2: “Life changes”

Category (Subcategory)	Meaning units
Being diagnosed with diabetes (Comparison with other people with diabetes, relations to surrounding persons)	When [I] heard [I] have diabetes [it came] all at once […] I saw before me the people who get insulin […] if you travel anywhere you … it’s not so simple … (IP3)
	I [have] lots of mates who are seriously ill […] they’re injecting all the time […] and they live a perfectly good life (IP6)
	my sister always had to go for pedicure […] so I thought that would be the only positive thing about this, that you could get pedicure, but she didn’t think I needed that so nothing came of it (IP9)
	my ex and my children’s mother think […] you shouldn’t be reading and thinking too much (IP7)
	the only one who knows [about my diagnosis] is my dietician […] and a close workmate […] who [also] has diabetes (IP9)
	but there’s also a witch-hunt on […] people who are overweight or obese [or] smoke [or] drink a lot […] often their own fault because that’s something you can influence […], and the debate isn’t always so nice […] they demand a bit of the patients […], they don’t feel sorry for them (IP9)
Therapeutic treatment (Non-pharmacological and pharmacological treatment)	life [hasn’t] changed much, except that I’ve stopped … a lot of sweets and sugar in my coffee and lost seven kilos (IP1) it’s a bit hard [to change anything] such as now when I eat bread that I never liked […] but now you’re not allowed to eat everything you want (IP4)
	I was quite good [about taking exercise] at first but, uh, well … I’ve maybe cut it down a bit and would maybe need … to walk a bit more (IP1)
	I find it very difficult to swallow tablets so my only thought was how will this go, but … it’s gone well (IP5)
	I take so many tablets that it doesn’t matter if I take more (IP6)
	[I worry about insulin] because then there’ll be no more flying (IP1)
	I saw before me the people who get insulin […] if you travel anywhere you … it’s not so simple … (IP3)
	I hate injections too, that’s another thing (IP9)
	as an adult I don’t think it [insulin] is such a big deal … the syringes are so fine today, it’s not so terrible (IP10)
Relation to health care (Expectations, trust)	[What I] expect of the doctor and the diabetic nurse is above all knowledge and that they are involved in research and development in the field (IP2)
	the diabetic nurse refers to the doctor when it comes to medication [and the doctor] refers to the doctor in the hospital … so that I don’t have any concerted point […] you feel rather alone [in the health service] (IP7)
	I’ve had really good [help from health care], they have a very good […] organization for this diabetes thing (IP9)
	I think the key word in all medication [is] the participating patient (IP2)
	it’s not the case that I phone and book an appointment [to discuss], if you look out in the waiting room it’s packed so you can’t always do it for reasons of availability so it a good thing that we have had some regular visits […] I appreciate that part (IP7)
The importance of knowledge (Obtaining supplementary information about diabetes, relating individually to the information)	I have a son who […] works in health care […] and he’s living with a doctor so I’ve had a bit of information there (IP1)
	[there] was a bit of researching on the internet about what this [the diagnosis] involves (IP7)
	but I think that if I am to accept a diabetes diagnosis that is chronic in character then I must accept and understand how my body functions […] I felt that I must make it my responsibility and start reading (IP7)

The *relation to surrounding persons* and their comments was very important. A common annoying notion was that the surrounding persons were interfering and had comments on how the interviewees should live their life. One interviewee expressed difficulties telling friends about the diagnosis. At the same time it was important to have someone to talk to, preferably other persons with diabetes, to share experiences and problems.

*“You have to shut your ears to some people, the people around you saying that I should go out for a walk, I should do this and that.” (P7)*

The **therapeutic treatment**, both the *non-pharmacological* and the *pharmacological*, changed the interviewees’ lives.

The *non-pharmacological treatment* consisted of dietary changes and physical activity. Concerning dietary changes there were a variety of experiences, for some difficult and a huge commitment, whereas the majority did not mention any great changes or problems. The challenge was changing a long-settled behavior, eating food you never liked and maintaining the changes over time. Personal responsibility was seen clearly by most interviewees. It could be an intense feeling of bad conscious or guilt towards society. Diabetes was caused by the interviewees’ overeating and now they burdened the society’s economy. Changing physical activity was also very difficult, even if personal responsibility was clearly felt. Some succeeded in long-term changes whereas the majority returned to old habits or did not manage to change their behavior at all.

Some succeeded in changing their behavior and kept the changes at least until the time of the interview whereas the majority returned sooner to old habits or did not manage to change their behavior at all.

The *pharmacological treatment* concerned oral medication and injection of insulin and the difference was huge for all interviewees. Oral medication was no problem for the majority, although some experienced skepticism or fear at the start. Overall, the need for drugs was accepted, especially by those already taking other medications; one more pill was no big deal. In contrast, need for insulin treatment in the future was seen as a huge threat, associated with prejudices and fear. The interviewees were afraid of injections and the possible consequences for daily life, such as hindrance for travel or performing favorite leisure-time activities. In any case, some of the interviewees concluded that if they had to comply they would manage and accept it.

*“If there is something I am thinking about then it’s how long I can manage on Metformin so that you don’t suddenly have to start injecting.” (P1)*

The **relationship to health care** was one of the central parts in the new life of the patients. The most important *expectations* on health care were updated knowledge, continuity of care and not being left alone. The majority of interviewees showed *trust in* their GP or the specialized nurse and felt actively involved in treatment and pointed out the importance of this. The patients do the basic work and health care provides support and planning.

The **importance of knowledge** was experienced by all interviewees. Some participants were content and received the necessary information from the health care staff even though it was sometimes difficult to come into contact, especially with the GP. The majority, however, needed *to obtain supplementary information about diabetes* in different ways. Several consulted people in their family. There were different opinions about obtaining information from the internet, which was seen as very positive by some whereas others would never use the internet for information on diseases.

*“I never google diseases […] I call my brother who is a medical doctor […] I think it is stupid to try to diagnose yourself and suddenly you have got a whole host of diseases […] and then you start reading about it and then you start feeling inside your body, no, that’s nothing for me.” (P10)*

The participants *related individually to the information* obtained. Some individuals were hardly affected at all by the information. Others related the information very much to themselves, they felt pressure on them and used it to plan for individual changes such as weight reduction.

### Concerns about the future (Table 6)

Even though the participants in general expressed few worries about the future, several areas were mentioned.

**Table 6:** Categories and examples of meaning units for theme 3 “Concerns about the future”

Category (Subcategory)	Meaning units
Family (Heredity, taking care of their family)	if it’s my children who are affected, that’s what you think, when you get a disease then maybe they’ll inherit this (IP4)
	you get a bit worried because you’ve got a disease that will be with you the whole of your life and when you have children and a family you think a little extra (IP4)
Functional disabilities (Physical complications and their consequences)	it would be unfortunate if my sight was affected because I can only see in one eye as it is today […] if that gets worse I’ll be blind in practice. I read quite a lot, so if my sight deteriorates even more it would mean a much much worse life […] I have devoted much of my activity to reading, watching television, keeping informed in general […] So if the sharpness of vision got lost I would be isolated and it would be very serious if that happened (IP2)
	what I worry about, I suppose […] my heart, I that think it has had to work rather hard and maybe it will give up some day […] if it’s damaged it’s damaged […] then I can’t influence it so much (IP9)
	foot ulcers are troublesome […] so I wouldn’t want that, and I don’t want to go blind either […] but foot ulcers are probably what I’m most afraid of, well, not afraid, but I don’t want them (IP10) the only thing that worries me was that I would have to stop flying (IP1)
Attitudes towards control and risk (Need for control, wanting to know about risks of future complications)	[I] measure quite often at home, it’s because I want to be in control to see how it develops (IP2)
	the same as when I repair a car, for example … how long will I keep the car I’ve changed to, so you sometimes have to get a checkup (IP3)
	It’s good if you have knowledge […] about what you can expect and with that what you should be observant of and react to so that you can get care early, that’s important. (IP7)
	I think it’s better not to know [exactly what happens] […] I had a serious brain hemorrhage when I was 35 and if I’d known that before it would have been terrible (IP10)

There were worries for the **family**, both that their children could suffer from diabetes because of *heredity* but also that they would not be able *to take care of their family* in the future if they became too ill.

Spontaneously, the interviewees expressed few worries about what is happening inside the body, leading to possible **functional disabilities** in the future. When asked specifically about *physical complications and their consequences* they expressed fear that the feet, the heart and especially the eyes would be affected and concerns about restrictions in their daily life. They were especially worried about not being able to read, watch television or drive a car or not being able to get along on their own and needing the help of others.

There were different **attitudes towards control and risk.** Whereas some individuals showed a great *need for control*, for example by frequently measuring their blood glucose levels at home, others did not express such needs at all. Similarly, patients did not agree on *wanting to know about the risk of future complications*. The majority wanted to know what could happen in the future and what to expect in order to protect themselves and be observant to signs and symptoms. Others, however, said that not knowing was better, both concerning complications and about the risk of dying earlier than expected.

*“If you could diagnose a base level and then know the progress, […] a way to see that if it is like that after thirty-six months you usually see this kind of deterioration and so on, so that […] you have something to be prepared for … as an engineer it would have been nice to know … then you would have known when it’s time to change the car … but unfortunately I can’t change my body.” (P7)*

## Discussion

Whereas one can find a lot of studies on interviews with diabetes patients who have had their diagnosis for a long time, published qualitative studies on newly diagnosed diabetes patients are harder to find.

In a Scottish study using in-depth interviews of 40 newly diagnosed patients with type 2 diabetes, many interviewees showed uncertainty about the diagnosis. Most wanted the diagnosis confirmed by specialists at the hospital before they felt confident about making lifestyle changes [10]. At a follow-up those patients expressed a need for primary care professionals who had expertise in diabetes care, had more dedicated time and were more accessible than general practitioners [22]. An English study conducting 30 semi-structured interviews revealed a diversity in the quality of motivation, both between and within individuals over time, talking even about guilt and experiences of frustration [23]. In a US study of 16 adults using questionnaires and cognitive mapping with Post-It notes the predominant fields of interest were food, negative emotions, and the risks and complications of diabetes, with the focus mainly on self-management and very little on medication [24]. Those studies showed the complexity of the patients’ thoughts at diabetes diagnosis with the main focus on lifestyle changes rather than on medication, which goes along with our own findings.

### Reaction at diagnosis

Surprisingly, the majority of the interviewees did not express many feelings or had made no important changes in life after their diabetes diagnosis. As patients with type 2 diabetes mellitus die of cardiovascular disease at rates 2–4 times higher than patients without diabetes [3] physicians should regard a diabetes diagnosis as very important and having great impact on the patients’ future health and risk of complications. Even though this has not been studied before we assume that physicians could expect patients to react more strongly.

Regarding the modest reaction of the majority of the participants in our study after being diagnosed we did not find any studies with which to compare this quite astonishing result, which might be explained by the fact that in most studies the patients were not newly diagnosed but had already been living with diabetes a long time.

The modest reaction of the patients in our study can partially be explained by the fact that diabetes was diagnosed at an early stage, often at annual checkups for other diseases. According to a recent Danish study [25] one-third of newly or recently diagnosed type 2 diabetes patients present a likely diabetic complication at disease onset, but it is not even sure if those patients had symptoms of the complications. Thus most patients newly diagnosed with diabetes can perceive diabetes as silent and with few or no symptoms.

In addition, some of the interviewees had almost been waiting for the diagnosis and were not surprised when being informed. They had seemingly already accepted their fate, which could explain their modest reaction.

### Lifestyle changes

*To give dietary advice with the aim of improving the diet and* trying to increase the level of physical activity is an important part of the diabetes check-up in Sweden, especially in the meeting with the diabetes nurse. According to the National Guidelines [8] the check-ups include information, motivational talks and even the possibility to write a prescription for physical activity to facilitate for the patient to become more active [26]. Regarding the diet, an adapted energy intake and improved eating habits are important interventions to stabilise blood glucose and to reach weight loss if needed, using the National Board of Health and Welfare’s guidance Diet in Diabetes [27] as a complement in the consultation.

While some interviewees found it easy to make changes in diet and physical activity, the majority described obstacles and especially the risk of returning to previous lifestyle. The difficulties in long-term lifestyle changes are well-known problems, described in several studies [28–30], especially concerning physical activity [31]. In the current interviews the reasons for this were varied, making it difficult to draw general conclusions about which way to support the patient would be best. Other studies [28] describe three valuable and effective fields for long-term effects in lifestyle changes: to increase the length and to intensify treatment, to identify “high-risk” situations and barriers, and to involve friends or family and to work in groups. According to the authors [28], this can be combined with Motivational Interviewing (MI). On the other hand the *Swedish Agency for Health Technology Assessment and Assessment of Social Services (SBU) reported that there is not sufficient evidence that MI gives additional effect to changing habits concerning dietary or physical activity* [32]*, while they point out the importance of group interventions.*

The difficulty of getting patients to feel engaged in their diabetes and follow the physicians’ recommendations is a well-known problem all over the world. It is due to multiple complex factors not easy to understand [33]. Important factors are the patients’ knowledge about diabetes, beliefs and attitudes and the relationship with health-care professionals [34, 35]. It is common that doctors and patients don’t share the same point of view about what optimal treatment of diabetes looks like [36]. Lifestyle changes for the patient can be extensive and challenging and it is important for the physicians to know the patients’ emotional obstacles and experiences, in order to achieve a successful treatment [19].

### Medical treatment

Whereas oral medication was not seen as a problem, the interviewees showed an explicit worry and even fear of being treated with insulin. Although this has been described before in previous studies [37] it was somehow surprising that almost all interviewees expressed this fear. This is very important information for both physicians and nurses when starting to discuss insulin treatment with the patient. The fear was based partially on prejudices which have to be addressed.

### Complications

Most qualitative studies including patients with diabetes address experiences and observations in patients already suffering from complications but there are not, as far as we know, studies on the patients’ thoughts about the risk of future complications.

In our study visual impairment and blindness were the main complications the interviewees feared. These are not particularly common complications today, nor are they what physicians focus most on. The major part of the annual checkup is instead focused on risk factors (high blood pressure and hyperlipidemia) increasing the risk of complications from the heart, the kidneys and the brain, trying to prevent mainly macrovascular complications and kidney and heart diseases.

In newly diagnosed patients with diabetes type 2 who were followed in Sweden with retinal screening for 10 years, 96% of the patients’ visual acuity was good enough for driving-license and only one of the 548 participants was blind as a consequence of diabetes [38].

A British Study showed that diabetic retinopathy or maculopathy are no longer the leading cause of certifiable blindness among working age adults in England and Wales, probably due to the introduction of nationwide diabetic retinopathy screening programs and improved glycogenic control [39]. The situation seems to be similar in the rest of Europe, Northern America and most parts of Asia. On the other hand a study from Malawi showed a prevalence of sight-threatening diabetic retinopathy four to ten times higher than in Europe, probably due to late diagnosis of diabetes, poor access to health services and inadequate drug supply, as well as comorbidity [40].

### Information and communication with the health care staff

The interviewees differed in their way of retrieving and accepting information. Some were satisfied with the information they received from health care whereas others wanted to know more and searched actively for more information at an early stage. This is important knowledge for health care, especially for the first meetings with the nurses. The patients have to be approached individually after expressing their personal wishes and preferences.

It is well known that the agenda of the physicians and patients can differ and that good doctor-patient communication is essential [41], not least in consultations concerning chronic diseases such as diabetes. Doctors and patients have different approaches and thoughts about diabetes and its treatment and control, making communication more difficult. In 25% of diabetes consultations not all the patients’ concerns were addressed [42]. The physicians are more focused on laboratory test results and guidelines than on understanding the patients’ point of view and treatment goals. This leads to frustration and obstacles in doctor-patient communication [43]. In the National Guidelines for Diabetes Care provided by the National Board of Health and Welfare the focus is on measured values and quantitative quality indicators while only a short chapter addresses the communication with the patients and patients’ own involvement [8].

The experiences and observations the interviewees expressed in the current study were not homogeneous, as has been previously been shown [33], making it difficult to generalize about how communication with a patient with diabetes should be conducted, apart from the importance of individualizing and being aware of the different points of view [36]. To maintain the patient’s trust in health care is also a central issue [34].

The current study provides interesting findings about what patients especially focus on concerning their diabetes, which can be used to improve doctor-patient and nurse-patient communication. Physicians might think more about preventing myocardial infarction, kidney disease or stroke while patients are more focused on practical changes in their daily life such as not being able to travel, to drive a car, to practice their favorite leisure-time activities or to be in need of help from others.

Even though most of the interviewees wanted to know about long-term complications of diabetes, it is important to know that not all want this information. For some it meant a decline in quality of life if they were conscious about and confronted with what complications might happen. This is essential to think of when informing about possible complications. This is especially interesting because even the current consensus report from the American Diabetes Association and the European Association for the Study of Diabetes focuses on a patient-centered approach and individual treatment goals and strategies [44].

Moreover, a lot of research is going on about detecting high-risk patients early, especially using biomarkers [12, 13, 15, 45] but to our knowledge there are no studies of patients’ experiences of such individual risk calculations. This makes the current study, showing the respondents’ thoughts about risk and complications, important.

#### Strengths/weaknesses

Studies on newly diagnosed patients with diabetes are overall hard to find in the literature, so the current study fills a gap. Our choice to interview the patients within 12 months after being diagnosed with diabetes was a strength of the study as the respondents had had time to overcome the distress and surprise and were able to reflect on the diagnosis and develop thoughts for the future. At the same time the diagnosis was still fresh enough to make it easy to recall the situation.

The analysis benefited from being conducted by more than one researcher [21], the current interviews were analyzed by a team consisting of different professions, two GPs, MP and KBB and a behavioral scientist, ELS which creates a wider analytic frame. The interviewees had different social backgrounds and nationalities, making it possible to receive information from a variety of patients with diabetes. We also performed individual interviews giving the interviewees the possibility to speak openly, even about delicate or familiar areas touching sensitive feelings. On the other hand, there were some limitations. Compared to in-depth-interviews at least some interviews were rather short in time. This is a limitation which increases the risk that not all interviews elicited all the relevant information. Moreover the number of interviewees in the group was only 10, predominantly older males. The fact that the interviews were performed at a PHCC and not at a neutral place could be criticized. The respondent could act as a patient and the interviewer as a physician. At the same time it is an advantage that the interviewees felt comfortable and safe, and when asked they wanted to have the interviews at the PHCC.

## Conclusions

The majority of the interviewees with newly diagnosed diabetes did not spontaneously express strong feelings, nor had they experienced important changes in life regarding their diabetes diagnosis. On the other hand they expressed a large variety of thoughts and reactions concerning the diagnosis, from surprise and denial to neutral and acceptance. When asked, nearly all were concerned about the consequences for daily life and the future.

The point of view of the physician and patient might not focus on the same area, which can be an obstacle to communication. The results of this study might help to focus doctor-patient communication on issues highlighted by the patients, at the same time having in mind the importance of individualizing the information and recommendations for each patient.

## Additional file

Additional file 1: Interview guide. (DOCX 13 kb)

## Abbreviations

GP: General Practitioner
IP: Interviewperson
LDL: Low-density lipoprotein
P: Participant
PHCC: Primary healthcare center

## Authors’ information

The first author, MP, is a GP at a Primary Health Care Center (PHCC) working on a regular basis with patients with type 2 diabetes. Moreover she is a PhD and has previously published three studies on biomarkers and the risk of cardiovascular complications in newly diagnosed type 2 diabetes patients.

The co-author KBB is a GP with long clinical experience of diabetes care working at a PHCC. Moreover she has a PhD and long experience both as a researcher and as a tutor with many publications on hypertension and diabetes mellitus.

The co-author ELS is a behavioral scientist. She has a PhD and long experience of qualitative methods, both as a researcher and as a tutor.
