# Supplementary material for: “I have got diabetes!” – interviews of patients newly diagnosed with type 2 diabetes
Source: BMC Endocr Disord. 2019 May 24;19:53. doi: 10.1186/s12902-019-0380-5 (PMC6534850; doi:10.1186/s12902-019-0380-5)
Supplement: Supplementary file 1 — Interview guide. (DOCX 13 kb) [file 12902_2019_380_MOESM1_ESM.docx]

**Interviewguide to the project**

**“I have got diabetes!” –**

**interviews of patients newly diagnosed with type 2 diabetes**

**General information:** Thank the participant, present the purpose of the interview, presentation of the interviewer, explain the participant’s anonymity, his right to cancel the interview and the interview process

**Participants:** sex, age, born outside of Sweden / Europe, when was diabetes diagnosed?

**General open entry question:**

What is your experience and your thoughts about having being diagnosed with diabetes? What does it mean to you?

**Different fields:**

- Experiences concerning information given and how it has been given

Experiences of the disease and its treatment?

Probing questions:
Do you know why you need your medicines?

Has your doctor (or someone else) explained the medicine to you?

Did you get any new medication when you were diagnosed with diabetes? How did you feel about that?

Do you have confidence in your doctor / your diabetes nurse?
Do you feel yourself involved in your treatment?

- Thoughts about risk and complications of the disease

Probing questions:
How do you think about the risks and complications of diabetes?

Are you afraid of complications?

- Engagement and motivation to lifestyle changes

Probing questions:

How did life change since you got your diabetes diagnosis?

What was the biggest change? What was easiest?

Is there something else you would like to add or to conclude?

Do you have any questions?

Finish. Thank the participant.
